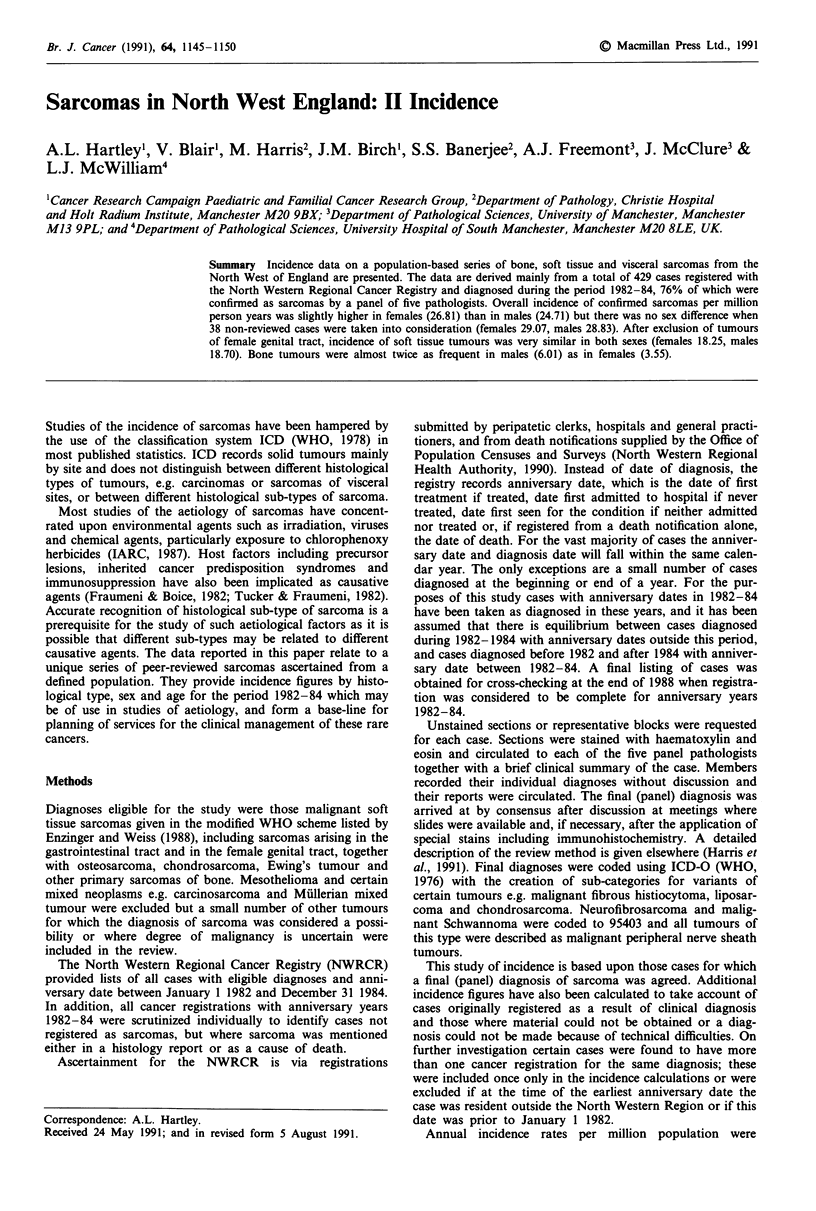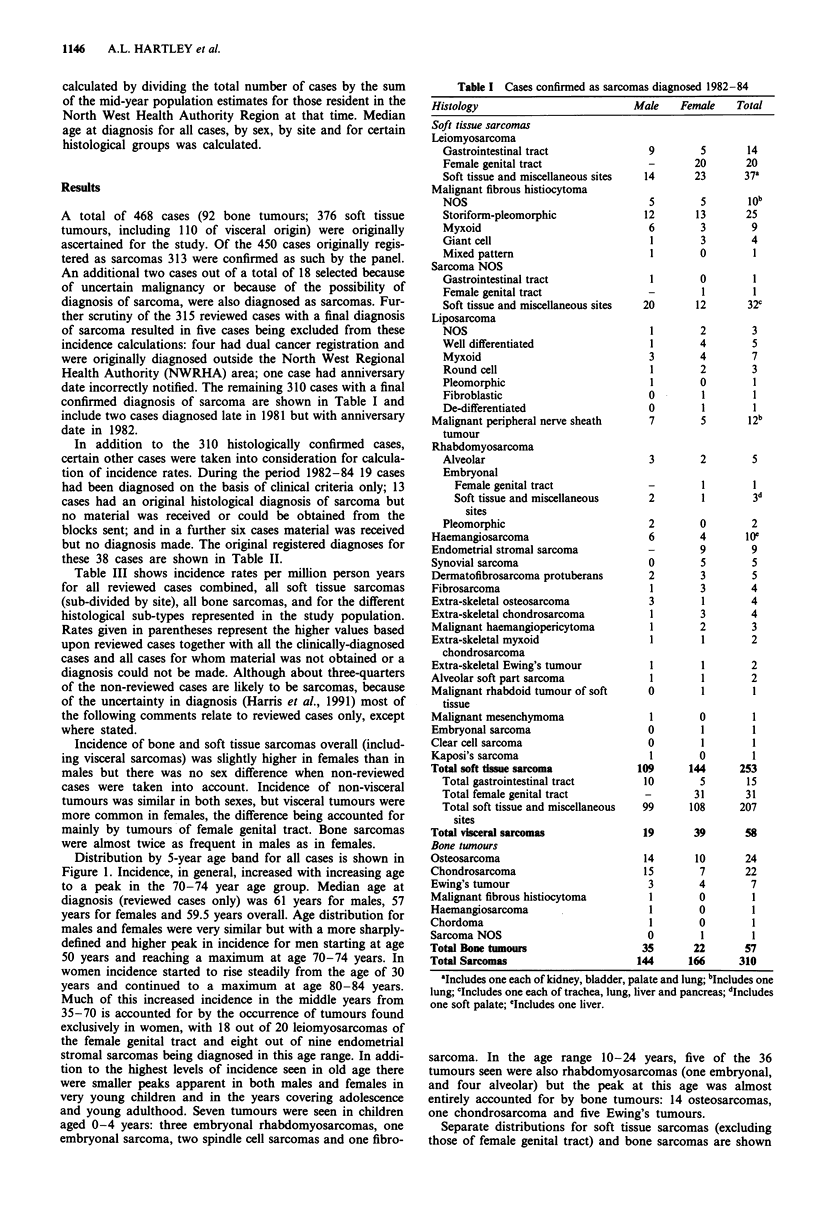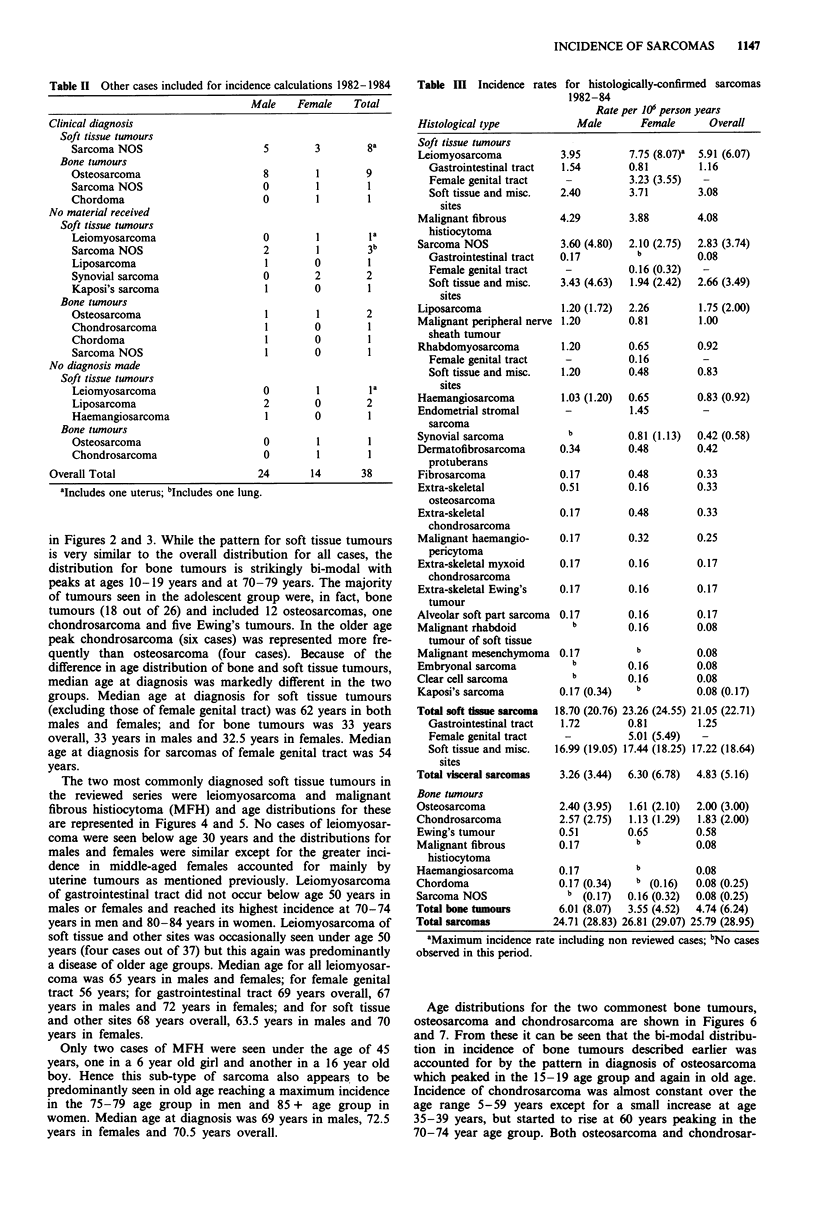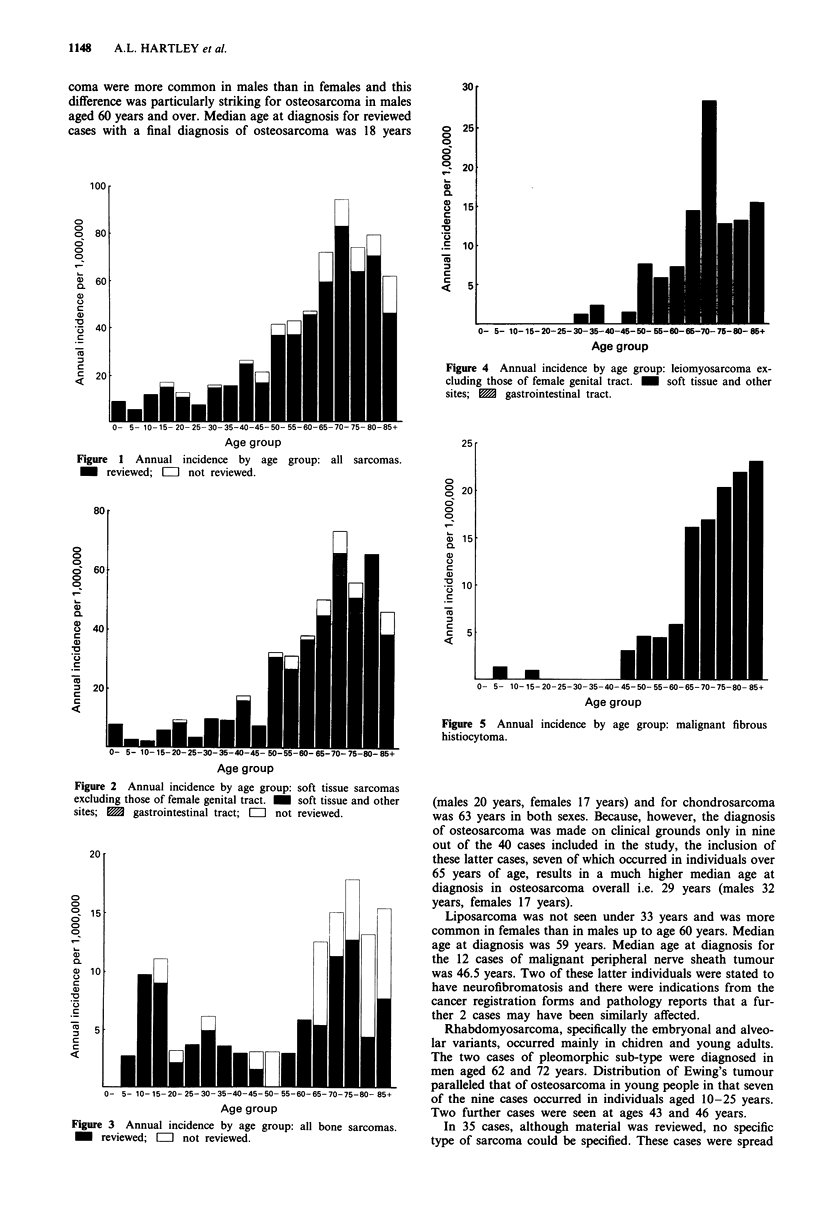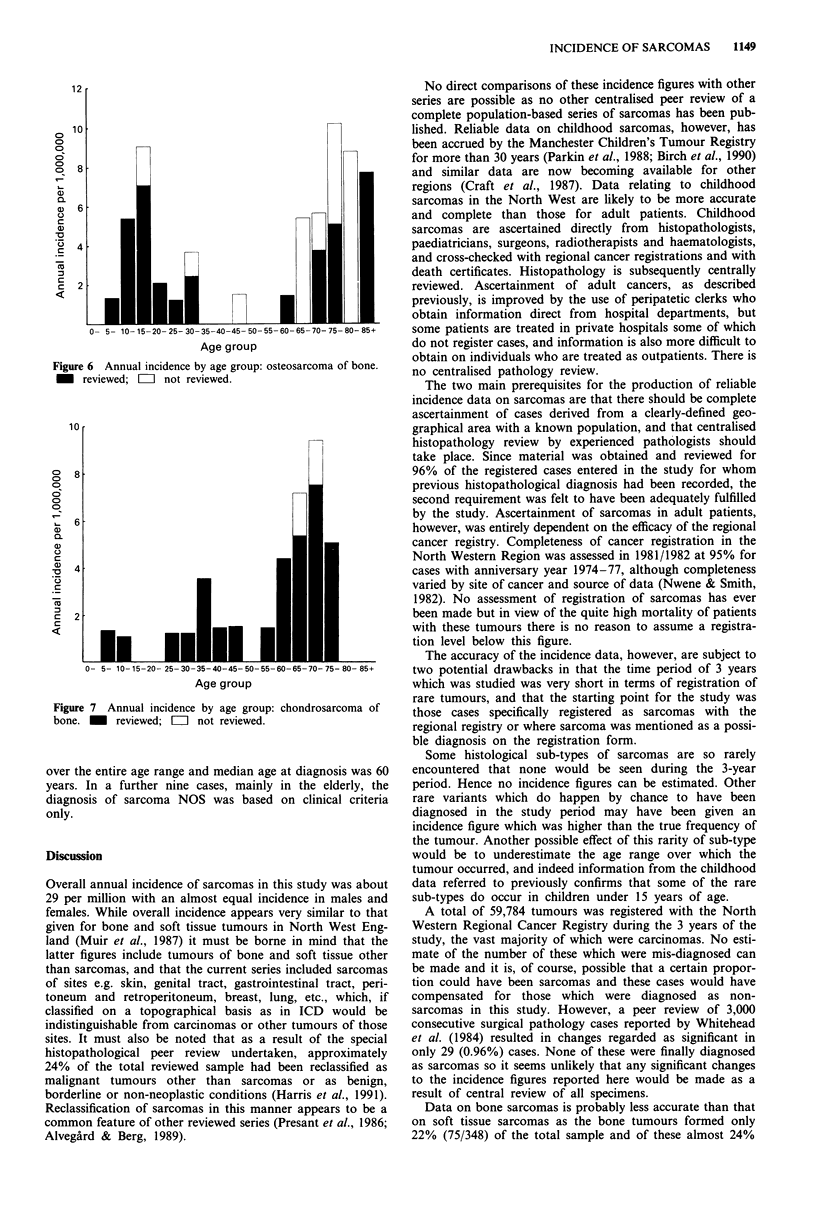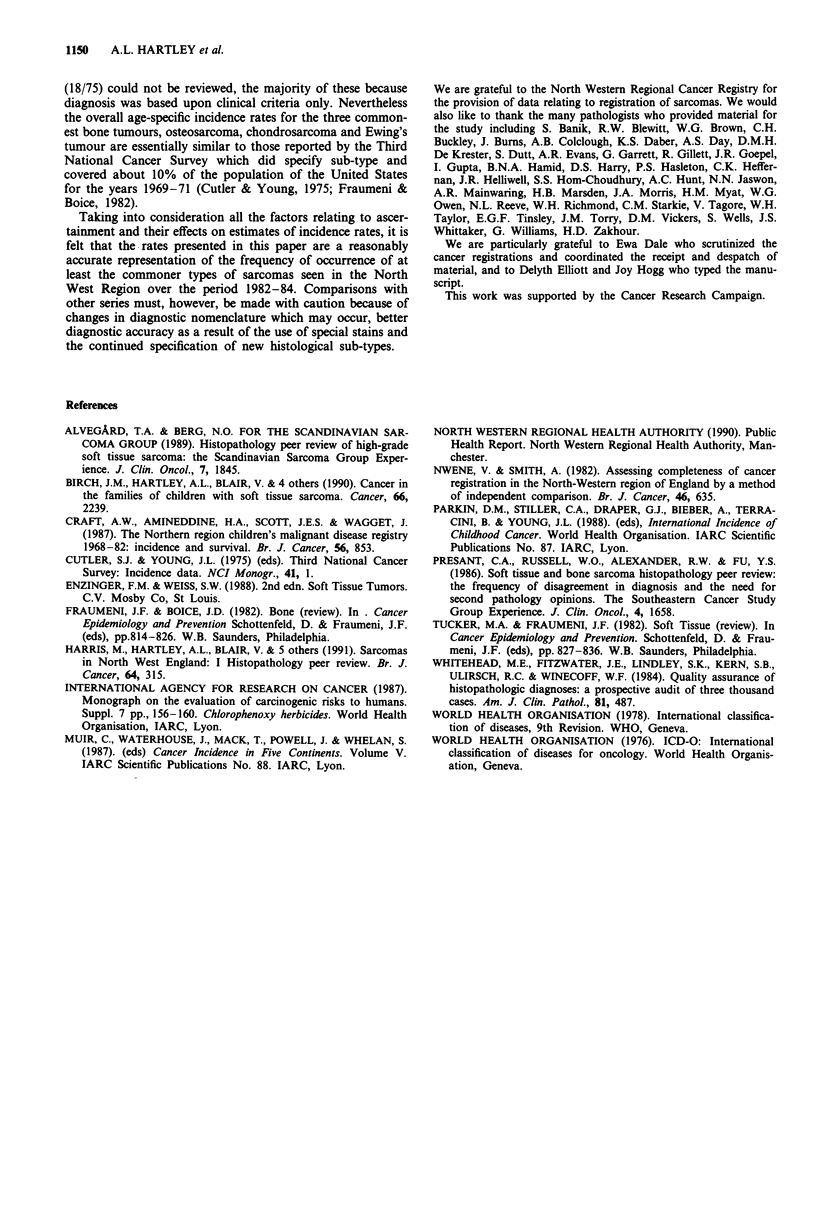# Sarcomas in north west England: II. Incidence.

**DOI:** 10.1038/bjc.1991.479

**Published:** 1991-12

**Authors:** A. L. Hartley, V. Blair, M. Harris, J. M. Birch, S. S. Banerjee, A. J. Freemont, J. McClure, L. J. McWilliam

**Affiliations:** Cancer Research Campaign Paediatric and Familial Cancer Research Group, UK.

## Abstract

Incidence data on a population-based series of bone, soft tissue and visceral sarcomas from the North West of England are presented. The data are derived mainly from a total of 429 cases registered with the North Western Regional Cancer Registry and diagnosed during the period 1982-84, 76% of which were confirmed as sarcomas by a panel of five pathologists. Overall incidence of confirmed sarcomas per million person years was slightly higher in females (26.81) than in males (24.71) but there was no sex difference when 38 non-reviewed cases were taken into consideration (females 29.07, males (28.83). After exclusion of tumours of female genital tract, incidence of soft tissue tumours was very similar in both sexes (females 18.25, males 18.70). Bone tumours were almost twice as frequent in males (6.01) as in females (3.55).


Br. J. Cancer (1991), 64, 1145–1150 © Macmillan Press Ltd., 1991

### Sarcomas in North West England: II Incidence

A.L. Hartley[1], V. Blair[1], M. Harris[2], J.M. Birch[1], S.S. Banerjee[2], A.J. Freemont[3], J. McClure[3] & L.J. McWilliam[4]

[1]*Cancer Research Campaign Paediatric and Familial Cancer Research Group,* [2]*Department of Pathology, Christie Hospital and Holt Radium Institute, Manchester M20 9BX;* [3]*Department of Pathological Sciences, University of Manchester, Manchester M13 9PL; and* [4]*Department of Pathological Sciences, University Hospital of South Manchester, Manchester M20 8LE, UK.*

**Summary** Incidence data on a population-based series of bone, soft tissue and visceral sarcomas from the North West of England are presented. The data are derived mainly from a total of 429 cases registered with the North Western Regional Cancer Registry and diagnosed during the period 1982–84, 76% of which were confirmed as sarcomas by a panel of five pathologists. Overall incidence of confirmed sarcomas per million person years was slightly higher in females (26.81) than in males (24.71) but there was no sex difference when 38 non-reviewed cases were taken into consideration (females 29.07, males 28.83). After exclusion of tumours of female genital tract, incidence of soft tissue tumours was very similar in both sexes (females 18.25, males 18.70). Bone tumours were almost twice as frequent in males (6.01) as in females (3.55).

Studies of the incidence of sarcomas have been hampered by the use of the classification system ICD (WHO, 1978) in most published statistics. ICD records solid tumours mainly by site and does not distinguish between different histological types of tumours, e.g. carcinomas or sarcomas of visceral sites, or between different histological sub-types of sarcoma.

Most studies of the aetiology of sarcomas have concentrated upon environmental agents such as irradiation, viruses and chemical agents, particularly exposure to chlorophenoxy herbicides (IARC, 1987). Host factors including precursor lesions, inherited cancer predisposition syndromes and immunosuppression have also been implicated as causative agents (Fraumeni & Boice, 1982; Tucker & Fraumeni, 1982). Accurate recognition of histological sub-type of sarcoma is a prerequisite for the study of such aetiological factors as it is possible that different sub-types may be related to different causative agents. The data reported in this paper relate to a unique series of peer-reviewed sarcomas ascertained from a defined population. They provide incidence figures by histological type, sex and age for the period 1982–84 which may be of use in studies of aetiology, and form a base-line for planning of services for the clinical management of these rare cancers.

### Methods

Diagnoses eligible for the study were those malignant soft tissue sarcomas given in the modified WHO scheme listed by Enzinger and Weiss (1988), including sarcomas arising in the gastrointestinal tract and in the female genital tract, together with osteosarcoma, chondrosarcoma, Ewing's tumour and other primary sarcomas of bone. Mesothelioma and certain mixed neoplasms e.g. carcinosarcoma and Müllerian mixed tumour were excluded but a small number of other tumours for which the diagnosis of sarcoma was considered a possibility or where degree of malignancy is uncertain were included in the review.

The North Western Regional Cancer Registry (NWRCR) provided lists of all cases with eligible diagnoses and anniversary date between January 1 1982 and December 31 1984. In addition, all cancer registrations with anniversary years 1982–84 were scrutinized individually to identify cases not registered as sarcomas, but where sarcoma was mentioned either in a histology report or as a cause of death.

Ascertainment for the NWRCR is via registrations submitted by peripatetic clerks, hospitals and general practitioners, and from death notifications supplied by the Office of Population Censuses and Surveys (North Western Regional Health Authority, 1990). Instead of date of diagnosis, the registry records anniversary date, which is the date of first treatment if treated, date first admitted to hospital if never treated, date first seen for the condition if neither admitted nor treated or, if registered from a death notification alone, the date of death. For the vast majority of cases the anniversary date and diagnosis date will fall within the same calendar year. The only exceptions are a small number of cases diagnosed at the beginning or end of a year. For the purposes of this study cases with anniversary dates in 1982–84 have been taken as diagnosed in these years, and it has been assumed that there is equilibrium between cases diagnosed during 1982–1984 with anniversary dates outside this period, and cases diagnosed before 1982 and after 1984 with anniversary date between 1982–84. A final listing of cases was obtained for cross-checking at the end of 1988 when registration was considered to be complete for anniversary years 1982–84.

Unstained sections or representative blocks were requested for each case. Sections were stained with haematoxylin and eosin and circulated to each of the five panel pathologists together with a brief clinical summary of the case. Members recorded their individual diagnoses without discussion and their reports were circulated. The final (panel) diagnosis was arrived at by consensus after discussion at meetings where slides were available and, if necessary, after the application of special stains including immunohistochemistry. A detailed description of the review method is given elsewhere (Harris *et al.*, 1991). Final diagnoses were coded using ICD-O (WHO, 1976) with the creation of sub-categories for variants of certain tumours e.g. malignant fibrous histiocytoma, liposarcoma and chondrosarcoma. Neurofibrosarcoma and malignant Schwannoma were coded to 95403 and all tumours of this type were described as malignant peripheral nerve sheath tumours.

This study of incidence is based upon those cases for which a final (panel) diagnosis of sarcoma was agreed. Additional incidence figures have also been calculated to take account of cases originally registered as a result of clinical diagnosis and those where material could not be obtained or a diagnosis could not be made because of technical difficulties. On further investigation certain cases were found to have more than one cancer registration for the same diagnosis; these were included once only in the incidence calculations or were excluded if at the time of the earliest anniversary date the case was resident outside the North Western Region or if this date was prior to January 1 1982.

Annual incidence rates per million population were

Correspondence: A.L. Hartley.
Received 24 May 1991; and in revised form 5 August 1991.

calculated by dividing the total number of cases by the sum of the mid-year population estimates for those resident in the North West Health Authority Region at that time. Median age at diagnosis for all cases, by sex, by site and for certain histological groups was calculated.

### Results

A total of 468 cases (92 bone tumours; 376 soft tissue tumours, including 110 of visceral origin) were originally ascertained for the study. Of the 450 cases originally registered as sarcomas 313 were confirmed as such by the panel. An additional two cases out of a total of 18 selected because of uncertain malignancy or because of the possibility of diagnosis of sarcoma, were also diagnosed as sarcomas. Further scrutiny of the 315 reviewed cases with a final diagnosis of sarcoma resulted in five cases being excluded from these incidence calculations: four had dual cancer registration and were originally diagnosed outside the North West Regional Health Authority (NWRHA) area; one case had anniversary date incorrectly notified. The remaining 310 cases with a final confirmed diagnosis of sarcoma are shown in Table I and include two cases diagnosed late in 1981 but with anniversary date in 1982.

In addition to the 310 histologically confirmed cases, certain other cases were taken into consideration for calculation of incidence rates. During the period 1982–84 19 cases had been diagnosed on the basis of clinical criteria only; 13 cases had an original histological diagnosis of sarcoma but no material was received or could be obtained from the blocks sent; and in a further six cases material was received but no diagnosis made. The original registered diagnoses for these 38 cases are shown in Table II.

Table III shows incidence rates per million person years for all reviewed cases combined, all soft tissue sarcomas (sub-divided by site), all bone sarcomas, and for the different histological sub-types represented in the study population. Rates given in parentheses represent the higher values based upon reviewed cases together with all the clinically-diagnosed cases and all cases for whom material was not obtained or a diagnosis could not be made. Although about three-quarters of the non-reviewed cases are likely to be sarcomas, because of the uncertainty in diagnosis (Harris *et al*., 1991) most of the following comments relate to reviewed cases only, except where stated.

Incidence of bone and soft tissue sarcomas overall (including visceral sarcomas) was slightly higher in females than in males but there was no sex difference when non-reviewed cases were taken into account. Incidence of non-visceral tumours was similar in both sexes, but visceral tumours were more common in females, the difference being accounted for mainly by tumours of female genital tract. Bone sarcomas were almost twice as frequent in males as in females.

Distribution by 5-year age band for all cases is shown in Figure 1. Incidence, in general, increased with increasing age to a peak in the 70–74 year age group. Median age at diagnosis (reviewed cases only) was 61 years for males, 57 years for females and 59.5 years overall. Age distribution for males and females were very similar but with a more sharply-defined and higher peak in incidence for men starting at age 50 years and reaching a maximum at age 70–74 years. In women incidence started to rise steadily from the age of 30 years and continued to a maximum at age 80–84 years. Much of this increased incidence in the middle years from 35–70 is accounted for by the occurrence of tumours found exclusively in women, with 18 out of 20 leiomyosarcomas of the female genital tract and eight out of nine endometrial stromal sarcomas being diagnosed in this age range. In addition to the highest levels of incidence seen in old age there were smaller peaks apparent in both males and females in very young children and in the years covering adolescence and young adulthood. Seven tumours were seen in children aged 0–4 years: three embryonal rhabdomyosarcomas, one embryonal sarcoma, two spindle cell sarcomas and one fibrosarcoma. In the age range 10–24 years, five of the 36 tumours seen were also rhabdomyosarcomas (one embryonal, and four alveolar) but the peak at this age was almost entirely accounted for by bone tumours: 14 osteosarcomas, one chondrosarcoma and five Ewing's tumours.

Separate distributions for soft tissue sarcomas (excluding those of female genital tract) and bone sarcomas are shown

**Table I** Cases confirmed as sarcomas diagnosed 1982–84

| *Histology* | *Male* | *Female* | *Total* |
|---|---|---|---|
| *Soft tissue sarcomas* | | | |
| Leiomyosarcoma | | | |
| Gastrointestinal tract | 9 | 5 | 14 |
| Female genital tract | – | 20 | 20 |
| Soft tissue and miscellaneous sites | 14 | 23 | 37[a] |
| Malignant fibrous histiocytoma | | | |
| NOS | 5 | 5 | 10[b] |
| Storiform-pleomorphic | 12 | 13 | 25 |
| Myxoid | 6 | 3 | 9 |
| Giant cell | 1 | 3 | 4 |
| Mixed pattern | 1 | 0 | 1 |
| Sarcoma NOS | | | |
| Gastrointestinal tract | 1 | 0 | 1 |
| Female genital tract | – | 1 | 1 |
| Soft tissue and miscellaneous sites | 20 | 12 | 32[c] |
| Liposarcoma | | | |
| NOS | 1 | 2 | 3 |
| Well differentiated | 1 | 4 | 5 |
| Myxoid | 3 | 4 | 7 |
| Round cell | 1 | 2 | 3 |
| Pleomorphic | 1 | 0 | 1 |
| Fibroblastic | 0 | 1 | 1 |
| De-differentiated | 0 | 1 | 1 |
| Malignant peripheral nerve sheath tumour | 7 | 5 | 12[b] |
| Rhabdomyosarcoma | | | |
| Alveolar | 3 | 2 | 5 |
| Embryonal | | | |
| Female genital tract | – | 1 | 1 |
| Soft tissue and miscellaneous sites | 2 | 1 | 3[d] |
| Pleomorphic | 2 | 0 | 2 |
| Haemangiosarcoma | 6 | 4 | 10[e] |
| Endometrial stromal sarcoma | – | 9 | 9 |
| Synovial sarcoma | 0 | 5 | 5 |
| Dermatofibrosarcoma protuberans | 2 | 3 | 5 |
| Fibrosarcoma | 1 | 3 | 4 |
| Extra-skeletal osteosarcoma | 3 | 1 | 4 |
| Extra-skeletal chondrosarcoma | 1 | 3 | 4 |
| Malignant haemangiopericytoma | 1 | 2 | 3 |
| Extra-skeletal myxoid chondrosarcoma | 1 | 1 | 2 |
| Extra-skeletal Ewing's tumour | 1 | 1 | 2 |
| Alveolar soft part sarcoma | 1 | 1 | 2 |
| Malignant rhabdoid tumour of soft tissue | 0 | 1 | 1 |
| Malignant mesenchymoma | 1 | 0 | 1 |
| Embryonal sarcoma | 0 | 1 | 1 |
| Clear cell sarcoma | 0 | 1 | 1 |
| Kaposi's sarcoma | 1 | 0 | 1 |
| **Total soft tissue sarcoma** | **109** | **144** | **253** |
| Total gastrointestinal tract | 10 | 5 | 15 |
| Total female genital tract | – | 31 | 31 |
| Total soft tissue and miscellaneous sites | 99 | 108 | 207 |
| **Total visceral sarcomas** | **19** | **39** | **58** |
| *Bone tumours* | | | |
| Osteosarcoma | 14 | 10 | 24 |
| Chondrosarcoma | 15 | 7 | 22 |
| Ewing's tumour | 3 | 4 | 7 |
| Malignant fibrous histiocytoma | 1 | 0 | 1 |
| Haemangiosarcoma | 1 | 0 | 1 |
| Chordoma | 1 | 0 | 1 |
| Sarcoma NOS | 0 | 1 | 1 |
| **Total Bone tumours** | **35** | **22** | **57** |
| **Total Sarcomas** | **144** | **166** | **310** |

[a]Includes one each of kidney, bladder, palate and lung; [b]Includes one lung; [c]Includes one each of trachea, lung, liver and pancreas; [d]Includes one soft palate; [e]Includes one liver.

**Table II** Other cases included for incidence calculations 1982–1984

| | *Male* | *Female* | *Total* |
|---|---|---|---|
| *Clinical diagnosis* | | | |
| *Soft tissue tumours* | | | |
| Sarcoma NOS | 5 | 3 | 8[a] |
| *Bone tumours* | | | |
| Osteosarcoma | 8 | 1 | 9 |
| Sarcoma NOS | 0 | 1 | 1 |
| Chordoma | 0 | 1 | 1 |
| *No material received* | | | |
| *Soft tissue tumours* | | | |
| Leiomyosarcoma | 0 | 1 | 1[a] |
| Sarcoma NOS | 2 | 1 | 3[b] |
| Liposarcoma | 1 | 0 | 1 |
| Synovial sarcoma | 0 | 2 | 2 |
| Kaposi's sarcoma | 1 | 0 | 1 |
| *Bone tumours* | | | |
| Osteosarcoma | 1 | 1 | 2 |
| Chondrosarcoma | 1 | 0 | 1 |
| Chordoma | 1 | 0 | 1 |
| Sarcoma NOS | 1 | 0 | 1 |
| *No diagnosis made* | | | |
| *Soft tissue tumours* | | | |
| Leiomyosarcoma | 0 | 1 | 1[a] |
| Liposarcoma | 2 | 0 | 2 |
| Haemangiosarcoma | 1 | 0 | 1 |
| *Bone tumours* | | | |
| Osteosarcoma | 0 | 1 | 1 |
| Chondrosarcoma | 0 | 1 | 1 |
| Overall Total | 24 | 14 | 38 |

[a]Includes one uterus; [b]Includes one lung.

in Figures 2 and 3. While the pattern for soft tissue tumours is very similar to the overall distribution for all cases, the distribution for bone tumours is strikingly bi-modal with peaks at ages 10–19 years and at 70–79 years. The majority of tumours seen in the adolescent group were, in fact, bone tumours (18 out of 26) and included 12 osteosarcomas, one chondrosarcoma and five Ewing's tumours. In the older age peak chondrosarcoma (six cases) was represented more frequently than osteosarcoma (four cases). Because of the difference in age distribution of bone and soft tissue tumours, median age at diagnosis was markedly different in the two groups. Median age at diagnosis for soft tissue tumours (excluding those of female genital tract) was 62 years in both males and females; and for bone tumours was 33 years overall, 33 years in males and 32.5 years in females. Median age at diagnosis for sarcomas of female genital tract was 54 years.

The two most commonly diagnosed soft tissue tumours in the reviewed series were leiomyosarcoma and malignant fibrous histiocytoma (MFH) and age distributions for these are represented in Figures 4 and 5. No cases of leiomyosarcoma were seen below age 30 years and the distributions for males and females were similar except for the greater incidence in middle-aged females accounted for mainly by uterine tumours as mentioned previously. Leiomyosarcoma of gastrointestinal tract did not occur below age 50 years in males or females and reached its highest incidence at 70–74 years in men and 80–84 years in women. Leiomyosarcoma of soft tissue and other sites was occasionally seen under age 50 years (four cases out of 37) but this again was predominantly a disease of older age groups. Median age for all leiomyosarcoma was 65 years in males and females; for female genital tract 56 years; for gastrointestinal tract 69 years overall, 67 years in males and 72 years in females; and for soft tissue and other sites 68 years overall, 63.5 years in males and 70 years in females.

Only two cases of MFH were seen under the age of 45 years, one in a 6 year old girl and another in a 16 year old boy. Hence this sub-type of sarcoma also appears to be predominantly seen in old age reaching a maximum incidence in the 75–79 age group in men and 85+ age group in women. Median age at diagnosis was 69 years in males, 72.5 years in females and 70.5 years overall.

**Table III** Incidence rates for histologically-confirmed sarcomas 1982–84

| | *Rate per 10⁶ person years* | | |
|---|---|---|---|
| *Histological type* | *Male* | *Female* | *Overall* |
| *Soft tissue tumours* | | | |
| Leiomyosarcoma | 3.95 | 7.75 (8.07)[a] | 5.91 (6.07) |
| Gastrointestinal tract | 1.54 | 0.81 | 1.16 |
| Female genital tract | – | 3.23 (3.55) | – |
| Soft tissue and misc. sites | 2.40 | 3.71 | 3.08 |
| Malignant fibrous histiocytoma | 4.29 | 3.88 | 4.08 |
| Sarcoma NOS | 3.60 (4.80) | 2.10 (2.75) | 2.83 (3.74) |
| Gastrointestinal tract | 0.17 | [b] | 0.08 |
| Female genital tract | – | 0.16 (0.32) | – |
| Soft tissue and misc. sites | 3.43 (4.63) | 1.94 (2.42) | 2.66 (3.49) |
| Liposarcoma | 1.20 (1.72) | 2.26 | 1.75 (2.00) |
| Malignant peripheral nerve sheath tumour | 1.20 | 0.81 | 1.00 |
| Rhabdomyosarcoma | 1.20 | 0.65 | 0.92 |
| Female genital tract | – | 0.16 | – |
| Soft tissue and misc. sites | 1.20 | 0.48 | 0.83 |
| Haemangiosarcoma | 1.03 (1.20) | 0.65 | 0.83 (0.92) |
| Endometrial stromal sarcoma | – | 1.45 | – |
| Synovial sarcoma | [b] | 0.81 (1.13) | 0.42 (0.58) |
| Dermatofibrosarcoma protuberans | 0.34 | 0.48 | 0.42 |
| Fibrosarcoma | 0.17 | 0.48 | 0.33 |
| Extra-skeletal osteosarcoma | 0.51 | 0.16 | 0.33 |
| Extra-skeletal chondrosarcoma | 0.17 | 0.48 | 0.33 |
| Malignant haemangio-pericytoma | 0.17 | 0.32 | 0.25 |
| Extra-skeletal myxoid chondrosarcoma | 0.17 | 0.16 | 0.17 |
| Extra-skeletal Ewing's tumour | 0.17 | 0.16 | 0.17 |
| Alveolar soft part sarcoma | 0.17 | 0.16 | 0.17 |
| Malignant rhabdoid tumour of soft tissue | [b] | 0.16 | 0.08 |
| Malignant mesenchymoma | 0.17 | [b] | 0.08 |
| Embryonal sarcoma | [b] | 0.16 | 0.08 |
| Clear cell sarcoma | [b] | 0.16 | 0.08 |
| Kaposi's sarcoma | 0.17 (0.34) | [b] | 0.08 (0.17) |
| **Total soft tissue sarcoma** | 18.70 (20.76) | 23.26 (24.55) | 21.05 (22.71) |
| Gastrointestinal tract | 1.72 | 0.81 | 1.25 |
| Female genital tract | – | 5.01 (5.49) | – |
| Soft tissue and misc. sites | 16.99 (19.05) | 17.44 (18.25) | 17.22 (18.64) |
| **Total visceral sarcomas** | 3.26 (3.44) | 6.30 (6.78) | 4.83 (5.16) |
| *Bone tumours* | | | |
| Osteosarcoma | 2.40 (3.95) | 1.61 (2.10) | 2.00 (3.00) |
| Chondrosarcoma | 2.57 (2.75) | 1.13 (1.29) | 1.83 (2.00) |
| Ewing's tumour | 0.51 | 0.65 | 0.58 |
| Malignant fibrous histiocytoma | 0.17 | [b] | 0.08 |
| Haemangiosarcoma | 0.17 | [b] | 0.08 |
| Chordoma | 0.17 (0.34) | [b] (0.16) | 0.08 (0.25) |
| Sarcoma NOS | [b] (0.17) | 0.16 (0.32) | 0.08 (0.25) |
| **Total bone tumours** | 6.01 (8.07) | 3.55 (4.52) | 4.74 (6.24) |
| **Total sarcomas** | 24.71 (28.83) | 26.81 (29.07) | 25.79 (28.95) |

[a]Maximum incidence rate including non reviewed cases; [b]No cases observed in this period.

Age distributions for the two commonest bone tumours, osteosarcoma and chondrosarcoma are shown in Figures 6 and 7. From these it can be seen that the bi-modal distribution in incidence of bone tumours described earlier was accounted for by the pattern in diagnosis of osteosarcoma which peaked in the 15–19 age group and again in old age. Incidence of chondrosarcoma was almost constant over the age range 5–59 years except for a small increase at age 35–39 years, but started to rise at 60 years peaking in the 70–74 year age group. Both osteosarcoma and chondrosarcoma were more common in males than in females and this difference was particularly striking for osteosarcoma in males aged 60 years and over. Median age at diagnosis for reviewed cases with a final diagnosis of osteosarcoma was 18 years (males 20 years, females 17 years) and for chondrosarcoma was 63 years in both sexes. Because, however, the diagnosis of osteosarcoma was made on clinical grounds only in nine out of the 40 cases included in the study, the inclusion of these latter cases, seven of which occurred in individuals over 65 years of age, results in a much higher median age at diagnosis in osteosarcoma overall i.e. 29 years (males 32 years, females 17 years).

Liposarcoma was not seen under 33 years and was more common in females than in males up to age 60 years. Median age at diagnosis was 59 years. Median age at diagnosis for the 12 cases of malignant peripheral nerve sheath tumour was 46.5 years. Two of these latter individuals were stated to have neurofibromatosis and there were indications from the cancer registration forms and pathology reports that a further 2 cases may have been similarly affected.

Rhabdomyosarcoma, specifically the embryonal and alveolar variants, occurred mainly in chidren and young adults. The two cases of pleomorphic sub-type were diagnosed in men aged 62 and 72 years. Distribution of Ewing's tumour paralleled that of osteosarcoma in young people in that seven of the nine cases occurred in individuals aged 10–25 years. Two further cases were seen at ages 43 and 46 years.

In 35 cases, although material was reviewed, no specific type of sarcoma could be specified. These cases were spread

**Figure 1** Annual incidence by age group: all sarcomas. ■ reviewed; □ not reviewed.

**Figure 2** Annual incidence by age group: soft tissue sarcomas excluding those of female genital tract. ■ soft tissue and other sites; ▨ gastrointestinal tract; □ not reviewed.

**Figure 3** Annual incidence by age group: all bone sarcomas. ■ reviewed; □ not reviewed.

**Figure 4** Annual incidence by age group: leiomyosarcoma excluding those of female genital tract. ■ soft tissue and other sites; ▨ gastrointestinal tract.

**Figure 5** Annual incidence by age group: malignant fibrous histiocytoma.

**Figure 6** Annual incidence by age group: osteosarcoma of bone. ■ reviewed; □ not reviewed.

**Figure 7** Annual incidence by age group: chondrosarcoma of bone. ■ reviewed; □ not reviewed.

over the entire age range and median age at diagnosis was 60 years. In a further nine cases, mainly in the elderly, the diagnosis of sarcoma NOS was based on clinical criteria only.

### Discussion

Overall annual incidence of sarcomas in this study was about 29 per million with an almost equal incidence in males and females. While overall incidence appears very similar to that given for bone and soft tissue tumours in North West England (Muir *et al.*, 1987) it must be borne in mind that the latter figures include tumours of bone and soft tissue other than sarcomas, and that the current series included sarcomas of sites e.g. skin, genital tract, gastrointestinal tract, peritoneum and retroperitoneum, breast, lung, etc., which, if classified on a topographical basis as in ICD would be indistinguishable from carcinomas or other tumours of those sites. It must also be noted that as a result of the special histopathological peer review undertaken, approximately 24% of the total reviewed sample had been reclassified as malignant tumours other than sarcomas or as benign, borderline or non-neoplastic conditions (Harris *et al.*, 1991). Reclassification of sarcomas in this manner appears to be a common feature of other reviewed series (Presant *et al.*, 1986; Alvegård & Berg, 1989).

No direct comparisons of these incidence figures with other series are possible as no other centralised peer review of a complete population-based series of sarcomas has been published. Reliable data on childhood sarcomas, however, has been accrued by the Manchester Children's Tumour Registry for more than 30 years (Parkin *et al.*, 1988; Birch *et al.*, 1990) and similar data are now becoming available for other regions (Craft *et al.*, 1987). Data relating to childhood sarcomas in the North West are likely to be more accurate and complete than those for adult patients. Childhood sarcomas are ascertained directly from histopathologists, paediatricians, surgeons, radiotherapists and haematologists, and cross-checked with regional cancer registrations and with death certificates. Histopathology is subsequently centrally reviewed. Ascertainment of adult cancers, as described previously, is improved by the use of peripatetic clerks who obtain information direct from hospital departments, but some patients are treated in private hospitals some of which do not register cases, and information is also more difficult to obtain on individuals who are treated as outpatients. There is no centralised pathology review.

The two main prerequisites for the production of reliable incidence data on sarcomas are that there should be complete ascertainment of cases derived from a clearly-defined geographical area with a known population, and that centralised histopathology review by experienced pathologists should take place. Since material was obtained and reviewed for 96% of the registered cases entered in the study for whom previous histopathological diagnosis had been recorded, the second requirement was felt to have been adequately fulfilled by the study. Ascertainment of sarcomas in adult patients, however, was entirely dependent on the efficacy of the regional cancer registry. Completeness of cancer registration in the North Western Region was assessed in 1981/1982 at 95% for cases with anniversary year 1974–77, although completeness varied by site of cancer and source of data (Nwene & Smith, 1982). No assessment of registration of sarcomas has ever been made but in view of the quite high mortality of patients with these tumours there is no reason to assume a registration level below this figure.

The accuracy of the incidence data, however, are subject to two potential drawbacks in that the time period of 3 years which was studied was very short in terms of registration of rare tumours, and that the starting point for the study was those cases specifically registered as sarcomas with the regional registry or where sarcoma was mentioned as a possible diagnosis on the registration form.

Some histological sub-types of sarcomas are so rarely encountered that none would be seen during the 3-year period. Hence no incidence figures can be estimated. Other rare variants which do happen by chance to have been diagnosed in the study period may have been given an incidence figure which was higher than the true frequency of the tumour. Another possible effect of this rarity of sub-type would be to underestimate the age range over which the tumour occurred, and indeed information from the childhood data referred to previously confirms that some of the rare sub-types do occur in children under 15 years of age.

A total of 59,784 tumours was registered with the North Western Regional Cancer Registry during the 3 years of the study, the vast majority of which were carcinomas. No estimate of the number of these which were mis-diagnosed can be made and it is, of course, possible that a certain proportion could have been sarcomas and these cases would have compensated for those which were diagnosed as non-sarcomas in this study. However, a peer review of 3,000 consecutive surgical pathology cases reported by Whitehead *et al.* (1984) resulted in changes regarded as significant in only 29 (0.96%) cases. None of these were finally diagnosed as sarcomas so it seems unlikely that any significant changes to the incidence figures reported here would be made as a result of central review of all specimens.

Data on bone sarcomas is probably less accurate than that on soft tissue sarcomas as the bone tumours formed only 22% (75/348) of the total sample and of these almost 24%

(18/75) could not be reviewed, the majority of these because diagnosis was based upon clinical criteria only. Nevertheless the overall age-specific incidence rates for the three commonest bone tumours, osteosarcoma, chondrosarcoma and Ewing's tumour are essentially similar to those reported by the Third National Cancer Survey which did specify sub-type and covered about 10% of the population of the United States for the years 1969–71 (Cutler & Young, 1975; Fraumeni & Boice, 1982).

Taking into consideration all the factors relating to ascertainment and their effects on estimates of incidence rates, it is felt that the rates presented in this paper are a reasonably accurate representation of the frequency of occurrence of at least the commoner types of sarcomas seen in the North West Region over the period 1982–84. Comparisons with other series must, however, be made with caution because of changes in diagnostic nomenclature which may occur, better diagnostic accuracy as a result of the use of special stains and the continued specification of new histological sub-types.

We are grateful to the North Western Regional Cancer Registry for the provision of data relating to registration of sarcomas. We would also like to thank the many pathologists who provided material for the study including S. Banik, R.W. Blewitt, W.G. Brown, C.H. Buckley, J. Burns, A.B. Colclough, K.S. Daber, A.S. Day, D.M.H. De Krester, S. Dutt, A.R. Evans, G. Garrett, R. Gillett, J.R. Goepel, I. Gupta, B.N.A. Hamid, D.S. Harry, P.S. Hasleton, C.K. Heffernan, J.R. Helliwell, S.S. Hom-Choudhury, A.C. Hunt, N.N. Jaswon, A.R. Mainwaring, H.B. Marsden, J.A. Morris, H.M. Myat, W.G. Owen, N.L. Reeve, W.H. Richmond, C.M. Starkie, V. Tagore, W.H. Taylor, E.G.F. Tinsley, J.M. Torry, D.M. Vickers, S. Wells, J.S. Whittaker, G. Williams, H.D. Zakhour.

We are particularly grateful to Ewa Dale who scrutinized the cancer registrations and coordinated the receipt and despatch of material, and to Delyth Elliott and Joy Hogg who typed the manuscript.

This work was supported by the Cancer Research Campaign.

### References

ALVEGÅRD, T.A. & BERG, N.O. FOR THE SCANDINAVIAN SARCOMA GROUP (1989). Histopathology peer review of high-grade soft tissue sarcoma: the Scandinavian Sarcoma Group Experience. *J. Clin. Oncol.*, **7**, 1845.

BIRCH, J.M., HARTLEY, A.L., BLAIR, V. & 4 others (1990). Cancer in the families of children with soft tissue sarcoma. *Cancer*, **66**, 2239.

CRAFT, A.W., AMINEDDINE, H.A., SCOTT, J.E.S. & WAGGET, J. (1987). The Northern region children's malignant disease registry 1968–82: incidence and survival. *Br. J. Cancer*, **56**, 853.

CUTLER, S.J. & YOUNG, J.L. (1975) (eds). Third National Cancer Survey: Incidence data. *NCI Monogr.*, **41**, 1.

ENZINGER, F.M. & WEISS, S.W. (1988). 2nd edn. Soft Tissue Tumors. C.V. Mosby Co, St Louis.

FRAUMENI, J.F. & BOICE, J.D. (1982). Bone (review). In . *Cancer Epidemiology and Prevention* Schottenfeld, D. & Fraumeni, J.F. (eds), pp.814–826. W.B. Saunders, Philadelphia.

HARRIS, M., HARTLEY, A.L., BLAIR, V. & 5 others (1991). Sarcomas in North West England: I Histopathology peer review. *Br. J. Cancer*, **64**, 315.

INTERNATIONAL AGENCY FOR RESEARCH ON CANCER (1987). Monograph on the evaluation of carcinogenic risks to humans. Suppl. 7 pp., 156–160. *Chlorophenoxy herbicides*. World Health Organisation, IARC, Lyon.

MUIR, C., WATERHOUSE, J., MACK, T., POWELL, J. & WHELAN, S. (1987). (eds) *Cancer Incidence in Five Continents*. Volume V. IARC Scientific Publications No. 88. IARC, Lyon.

NORTH WESTERN REGIONAL HEALTH AUTHORITY (1990). Public Health Report. North Western Regional Health Authority, Manchester.

NWENE, V. & SMITH, A. (1982). Assessing completeness of cancer registration in the North-Western region of England by a method of independent comparison. *Br. J. Cancer*, **46**, 635.

PARKIN, D.M., STILLER, C.A., DRAPER, G.J., BIEBER, A., TERRACINI, B. & YOUNG, J.L. (1988). (eds), *International Incidence of Childhood Cancer*. World Health Organisation. IARC Scientific Publications No. 87. IARC, Lyon.

PRESANT, C.A., RUSSELL, W.O., ALEXANDER, R.W. & FU, Y.S. (1986). Soft tissue and bone sarcoma histopathology peer review: the frequency of disagreement in diagnosis and the need for second pathology opinions. The Southeastern Cancer Study Group Experience. *J. Clin. Oncol.*, **4**, 1658.

TUCKER, M.A. & FRAUMENI, J.F. (1982). Soft Tissue (review). In *Cancer Epidemiology and Prevention*. Schottenfeld, D. & Fraumeni, J.F. (eds), pp. 827–836. W.B. Saunders, Philadelphia.

WHITEHEAD, M.E., FITZWATER, J.E., LINDLEY, S.K., KERN, S.B., ULIRSCH, R.C. & WINECOFF, W.F. (1984). Quality assurance of histopathologic diagnoses: a prospective audit of three thousand cases. *Am. J. Clin. Pathol.*, **81**, 487.

WORLD HEALTH ORGANISATION (1978). International classification of diseases, 9th Revision. WHO, Geneva.

WORLD HEALTH ORGANISATION (1976). ICD-O: International classification of diseases for oncology. World Health Organisation, Geneva.